# Supplementary material for: Identification and Tracking of Antiviral Drug Combinations
Source: Viruses. 2020 Oct 18;12(10):1178. doi: 10.3390/v12101178 (PMC7589631; doi:10.3390/v12101178)
Supplement: Supplementary file 1 [file viruses-12-01178-s001.pdf]

Supplementary information

Table S1. Compounds, their suppliers, and catalogue numbers.

Drug	CAS	Supplier	Purity (%)	Cat. #	MW	Formula
Brequinar	96187-53-0	Cayman Chemical	>98	24445	375	C23H15F2NO2
Emetine	7083-71-8	Cayman Chemical	>98	21048	481	C29H40N2O4 2HCl
Homoharringtonine	26833-87-4	Cayman Chemical	>98	14631	546	C29H39NO9
Lamivudine	134678-17-4	Selleckchem	>99	S1706	229	C8H11N3O3S
Monensin	22373-78-0	Cayman Chemical	≥98	16488	671	C36H61O11 • Na
Niclosamide	50-65-7	Dr. Ehrenstorfer	97	C15510000	327	C13H8Cl2N2O4
Nelfinavir	159989-65-8	Cayman Chemical	>98	15144	664	C32H45N3O4S CH3SO3H
Obatoclax	803712-79-0	MedChemExpress	99,7	HY-10969	413,49	$C_{21}H_{23}N_3O_4S$
Sofosbuvir	1190307-88-0	MedChemExpress	100	HY-15005	530	C22H29FN3O9P
Tenofovir	202138-50-9	Acros Organics	98	461250010	636	C19H30N5O10P C4H4O4
Vemurafenib	918504-65-1	MedChemExpress	99,8	HY-12057	490	$C_{23}H_{18}ClF_2N_3O_3S$

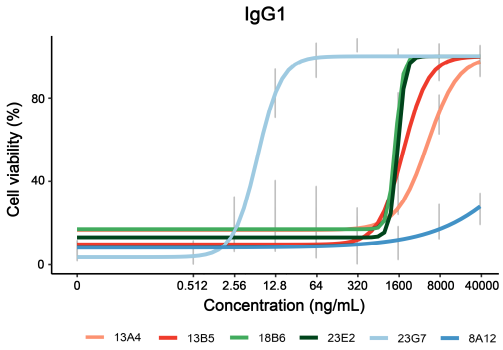

Fig. S1. The effect of recombinant antibodies from patients recovered from COVID-19 on virus-mediated death of Vero-E6 cells. (A) The HCoV-19/Norway/Trondheim-S15/2020 strain (moi 0.1) was incubated with indicated concentrations of recombinant antibodies derived from patients recovered from COVID-19 infections. The mixtures were added to Vero-E6 cells. Cell viability was measured after 72 h. Mean ± SD, n = 3.

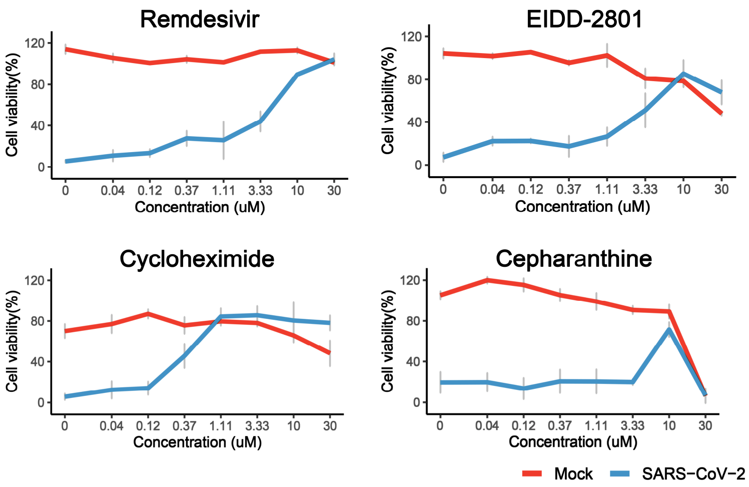

Fig. S2. Remdesivir, EIDD-2801, cycloheximide, and cepharanthine prevent the virus-mediated death of Vero-E6 cells. The cells were treated with increasing concentrations of a compound and infected with the HCoV-19/Norway/Trondheim-S15/2020 strain (moi, 0.1) or mock. After 72 h, cell viability was determined using CellTiter-Glo. Mean ± SD; n = 3.

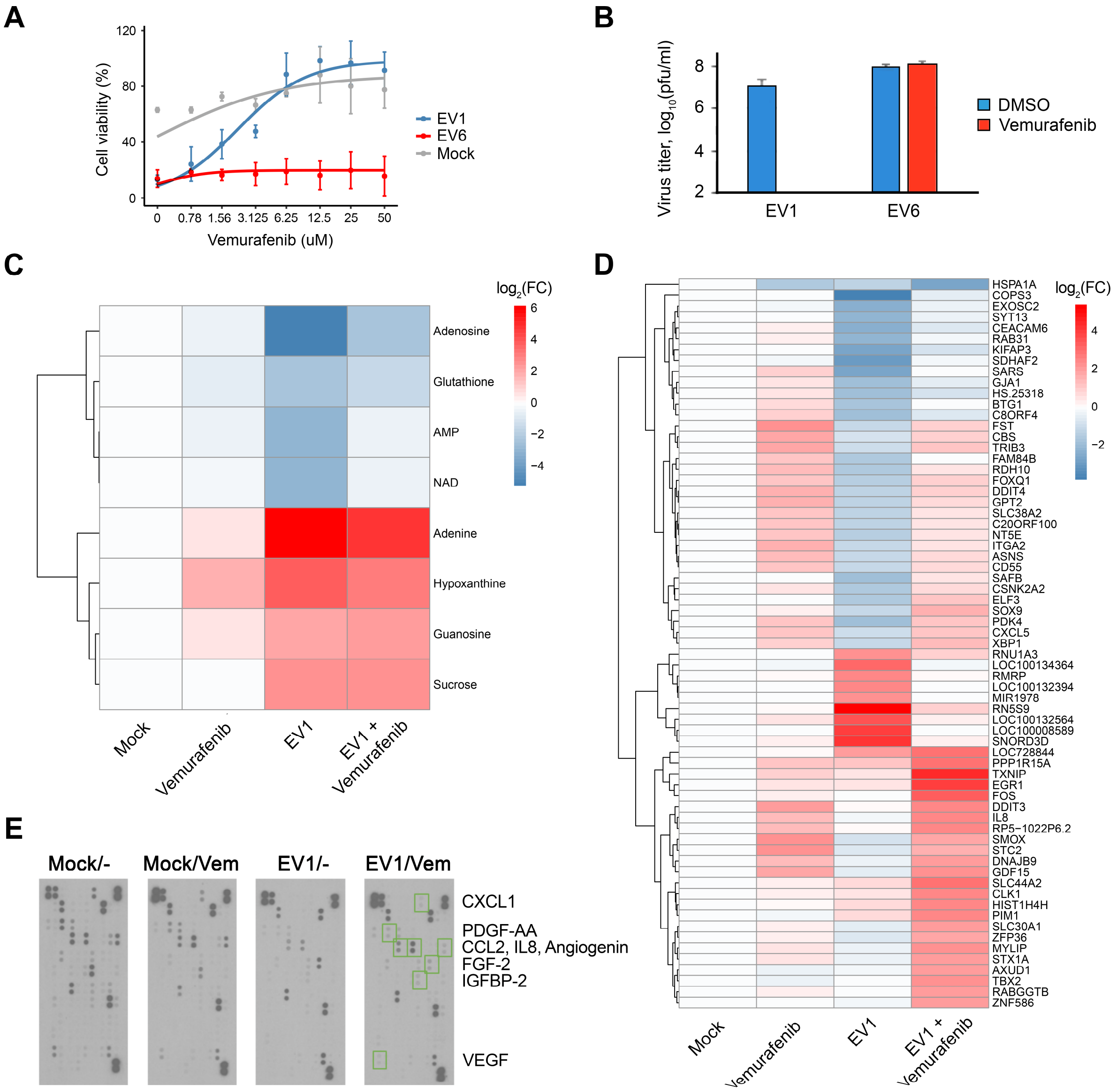

Fig. S3. Vemurafenib is a novel inhibitor of EV1 replication. (A) A549 cells were treated with increasing concentrations of vemurafenib or DMSO and infected with EV1 (moi, 0.1), EV6 (moi, 0.1) or mock. The viability of the cells was determined at 24 hpi with the Cell Titer Glo assay (mean ± SD; n = 3). (B) EV1 and EV6 produced in A549 cells treated with vemurafenib (10 μM) or DMSO was titered using plaque reduction assay, and the viral titers were calculated (mean ± SD; n = 3). (C) A549 cells were treated with 10 μM vemurafenib or non-treated, infected with EV1 (moi 0.1) or mock. After 24 h, cell culture supernatants were collected, and levels of 111 polar metabolites were determined using mass spectrometry. A heatmap of the most variable metabolites is shown (cut-off: log2FC > 1.5 and <−1.5). Rows represent metabolites, columns represent samples. Each cell is colored according to the log2-transformed values of samples, expressed as fold-change (FC) relative to the average of mock controls. Metabolites are ranked based on the FC (mean; n = 3). (D) A549 cells were treated with 10 μM vemurafenib or solvent and infected with EV1 (moi 0.1) or mock. At 8 hpi, the cells were collected, total RNA was extracted, and gene expression profiling was carried out. A heatmap of the most variable genes is shown (cut-off: log2FC > 1 and <−1). Rows represent gene symbols, columns represent treatments. Each cell is coloured according to the log2-transformed and quantile-normalized values of the samples, expressed as FC relative to the average of mock controls. (E) A549 cells were treated with 10 μM vemurafenib or non-treated, infected with EV1 (moi 0.1), or mock. After 24 h, cell culture media were collected, and relative levels of cytokines, chemokines, growth factors, and other soluble proteins were determined using a human cytokine array. Scanned array membranes are shown, and dots corresponding to affected cytokines are indicated.

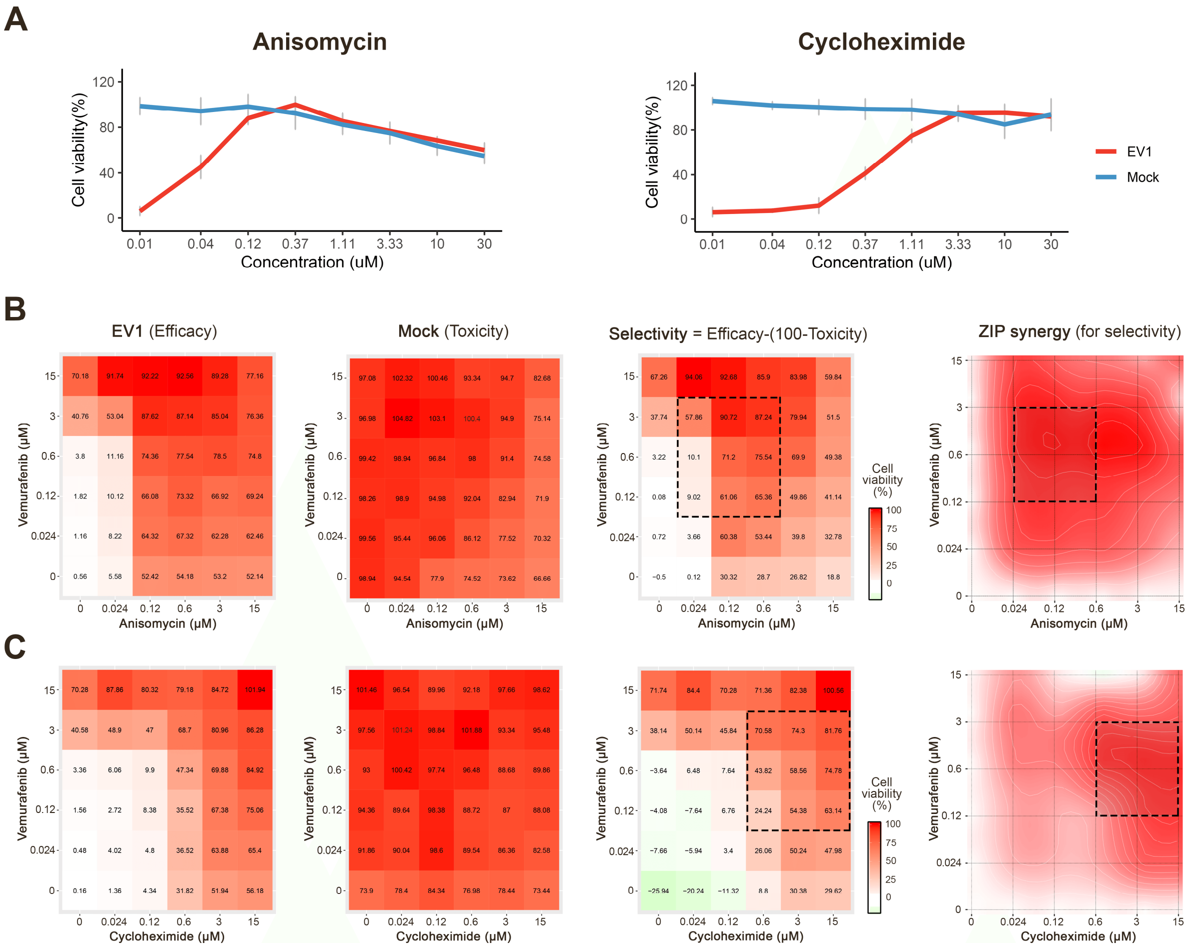

Fig. S4. Anisomycin and cycloheximide prevent the EV1-mediated death of Vero-E6 cells. (A). The cells were treated with increasing concentrations of a compound and infected with the EV1 (moi, 0.1) or mock. After 24 h, cell viability was determined using CellTiter-Glo assay. Mean ± SD; n = 3. (B,C, left panels) The interaction landscapes of drug combinations measured using a CTG assay on EV1- and mock-infected cells. (B,C, right panels) The interaction landscape showing selectivity and synergy of the drug combination.
